# [Retracted] Magnesium modulates the expression levels of calcification‑associated factors to inhibit calcification in a time‑dependent manner

**DOI:** 10.3892/etm.2025.12802

**Published:** 2025-01-20

**Authors:** Jinsheng Xu, Yaling Bai, Jingjing Jin, Junxia Zhang, Shenglei Zhang, Liwen Cui, Huiran Zhang

Exp Ther Med 9:1028–1034, 2015; DOI: 10.3892/etm.2015.2215

Following the publication of this article, a concerned reader drew to the Editor’s attention that, in Fig. 1 on p. 1030 showing the results of Alizarin red staining experiments, unexpected areas of similarity were identified in terms of the cellular patterns revealed within the data panels for three of the four different experiments portrayed in this figure, and also comparing between a pair of the panels.

After having conducted an internal investigation, the Editor of *Experimental and Therapeutic Medicine* has reached the conclusion that the overlapping sections of data shown in this figure were unlikely to have arisen by coincidence. Therefore, on the grounds of a lack of confidence in the integrity of these data, the Editor has decided that the article should be retracted from the publication. The authors were asked for an explanation to account for these concerns, but the Editorial Office did not receive a satisfactory reply. The Editor apologizes to the readership for any inconvenience caused, and thanks the interested reader for drawing this matter to our attention.

